# Regulation of gut microbiome by ketogenic diet in neurodegenerative diseases: A molecular crosstalk

**DOI:** 10.3389/fnagi.2022.1015837

**Published:** 2022-10-14

**Authors:** Shobana Kaviyarasan, Edmund Lee Chung Sia, Thaarvena Retinasamy, Alina Arulsamy, Mohd Farooq Shaikh

**Affiliations:** ^1^Clinical School Johor Bahru, Jeffrey Cheah School of Medicine and Health Sciences, Monash University Malaysia, Johor, Malaysia; ^2^Neuropharmacology Research Laboratory, Jeffrey Cheah School of Medicine and Health Sciences, Monash University Malaysia, Selangor, Malaysia

**Keywords:** ketogenic diet, high-fat diet, neurodegeneration, gut microbiota, brain-gut axis

## Abstract

The gut taxonomical profile is one of the contributory factors in maintaining homeostasis within the central nervous system (CNS). Of late, the efficacy of diet as a target of treatment, and how various dietary interventions may modulate gut microbiota differently have been an area of focus in research. The role of ketogenic diet (KD) in particular has been well-established in other diseases like intractable epilepsy due to its postulated effects on gut microbiome modulation, resulting in neuronal stability and prevention of epileptogenesis. Therefore, this systematic review aimed to critically evaluate the current available literature investigating the interplay between the three distinct entities: ketogenic diet, neurodegeneration, and gut microbiota, which may serve as a focus guide for future neurodegenerative diseases (ND) therapeutic research. A comprehensive literature search was performed on three databases; PubMed, Scopus, and Ovid Medline. A total of 12 articles were selected for critical appraisal, after subjecting to the inclusion and exclusion criteria in this study. The selected articles revealed that the hopes of KD as a treatment modality for ND are being ventured into as these individuals are said to acquire gut dysbiosis, primarily through increased colonization of phyla *Proteobacteria* and *Firmicutes*. Although positive effects including restoration of healthy gut microbes such as *Akkermansia Muciphilia* sp., improvement in cognitive functioning and decline in neuro-inflammatory markers were noted, this systematic review also depicted conflicting results such as decrease in alpha and beta species diversity as well as diminution of healthy gut commensals such as *Bifidobacteriace*. In addition, positive neuromodulation were also observed, notably an increase in cerebral blood perfusion to ventromedial hippocampal region *via* increased expression of eNOS and clearance of amyloid-beta proteins across the blood-brain-barrier *via* expression of p-glycoprotein. Neuroprotective mechanisms of ketogenic diet also included downregulation of mTOR expression, to prevention acceleration of pathological diseases such as Alzheimer's. Thus due to this conflicting/contrasting results demonstrated by ketogenic diet, such as a decline in gut species richness, diminution in beneficial microbes and decline cognition unless delivered in an intermittent fasting pattern, further studies may still be required before prior recommendation of a ketogenic diet therapeutic regime in ND patients.

## Introduction

Neurodegenerative diseases (ND) refer to a set of diverse pathological conditions associated with progressive structural or functional loss of neurons, which ultimately leads to irreversible neuronal death (Przedborski et al., 2003; Dugger and Dickson, 2017; Rekatsina et al., 2020). The epidemiology of ND varies according to each type of disease. The global prevalence of Alzheimer's disease (AD) appeared to be ~3% among those aged 65–74, with a drastic increase of 30% was observed amongst those aged 85 years and above (Harper, 2020). While the incidence of amyotrophic lateral sclerosis (ALS) was ~1 in 100,000, whereby this incidence rate increased with age (Checkoway et al., 2011). As for Parkinson's disease (PD), the prevalence rate was 2% in the population aged 65 and older (Checkoway et al., 2011). Extrapolation from these prevalence data suggested that the elderly population (age 65 years and above) may be the most vulnerable to ND development. Coupling this with the rampant projection of the aging population worldwide, in which the population was estimated to project to 1.5 billion individuals in the next 30 years (United Nations Department of Economic Social Affairs, 2020), ND may pose a significant impact or burden in the quality of life of patients and their caregivers, as well as on the country's healthcare and economy.

Besides epidemiology, each ND also has its distinct pathophysiology, such as extracellular aggregation of amyloid-beta plaques and intracellular tau depositions observed in AD, in contrast to accumulation of Lewy bodies in PD (Dugger and Dickson, 2017). However, the common intersection in all ND pathogenesis includes prolonged neuroinflammation, mitochondrial impairment, altered patterns of plasma amino acid levels and oxidative stress (Aquilani et al., 2020, 2022; Picca et al., 2020; Rekatsina et al., 2020). These pathological processes may be governed by an array of cellular and bio-molecular components, including the gut microbiome (Paoli et al., 2019). The gut microbiota consists of trillions of microorganisms, predominantly bacteria, and to a lesser extent, fungi, archaea and protozoa within the gastrointestinal tract (Paoli et al., 2019). The distinct pattern of the gut microbiome/microbiota in an individual may play a pivotal role in shifting the body's internal condition to either a favorable or adverse state. Some bacteria may possess beneficial anti-inflammatory properties (Tan et al., 2021), while others may promote widespread inflammation which may accelerate pathological disease processes, such as neurodegeneration (Tan et al., 2021).

**Figure 1:**
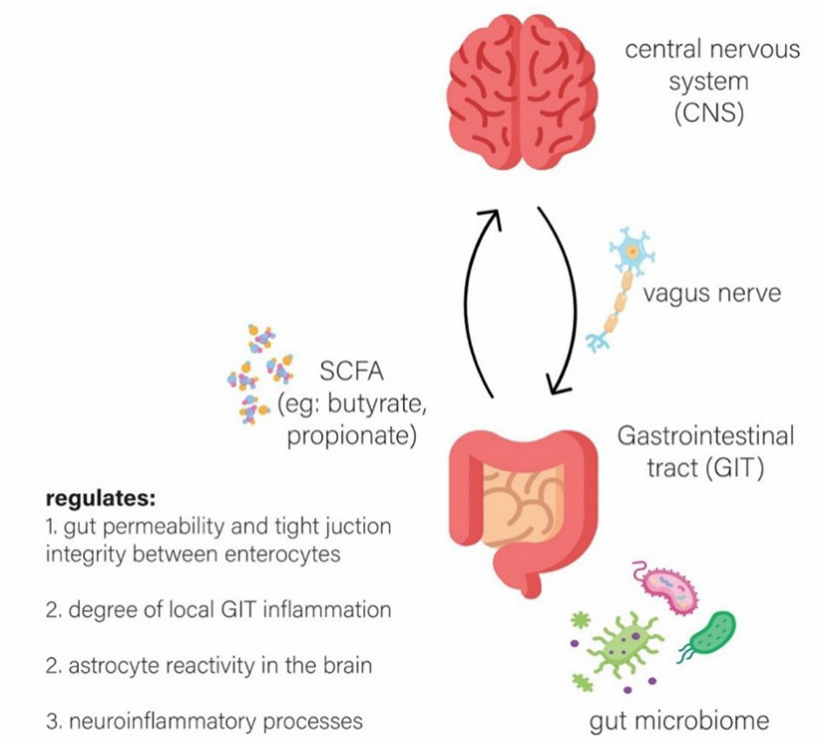
The bi-directional communication between the Microbiota, Gut and Brain (brain-gut axis).

Gut microbes may produce molecules of short-chain fatty acids (SCFAs) such as butyrate, propionate, and acetate, which may act as a relay to the neuroendocrine system and subsequently may regulate the degree of permeability of the blood brain barrier (BBB) and gut (Carabotti et al., 2015). These microbes may also modulate the degree of reactivity of astrocytes in neuroinflammation (Carabotti et al., 2015). Furthermore, the brain may modulate the enteric nervous system (ENS) *via* the parasympathetic system, through the vagus nerve (Carabotti et al., 2015), to ensure a smooth flow in gastrointestinal activity (Carabotti et al., 2015; Tan et al., 2021). Thus, this phenomenon may illustrate a two-way, dynamic interaction between the brain and the gut, known as the brain-gut axis, as illustrated in Figure 1, which may regulate homeostasis within the body (Rutsch et al., 2020). Therefore, this interaction may lead to the hypothesis that individuals with underlying gut dysbiosis, may result in a defective gut-brain axis communication, therefore leading to the progression of neurodegeneration (Carabotti et al., 2015; Rutsch et al., 2020).

Recent studies believed that the gut composition may be dynamic, and the introduction of specific dietary patterns such as the ketogenic diet was hypothesized to modulate the configurational state of the gut microbiota (Paoli et al., 2019; Koh et al., 2020). The macronutrient proportion of ketogenic diet included a very low carbohydrate content (not more than 10% of total caloric intake) and a high fat composition (~60%) (Masood et al., 2021), thus often interchangeably referred to as a high-fat diet as well. While this review has noted that ketogenic diet (very low carbohydrate) and high-fat diet (low carbohydrate) have been suggested in previous studies to have a distinctive impact on the gut microbiota (Ang et al., 2020), their differences have not been well established in the overall literature, with many researchers utilizing the terms interchangeably. Thus, this review has also employed the same approach.

Carbohydrate deficiency resulting from ketogenic diet may drive the body to a state of ketogenesis *via* fat metabolism, which was believed to positively modulate the gut microbiota and preserve optimal CNS functioning and performance (Koh et al., 2020). Ketogenic or high-fat diet have been hypothesized to improve gut dysbiosis and possibly attenuate neuroinflammation and neurodegeneration (Paoli et al., 2019).

As currently there has been very limited to no effective treatment available for most of the NDs, particularly devastating NDs like AD, PD, and ALS, exploring the therapeutic potential of ketogenic diet targeting the gut microbiota may bring new hope to ND patients and their caregivers. Therefore, this systematic review aimed to critically evaluate the current available literature investigating the interplay between the three distinct entities: ketogenic diet, neurodegeneration, and gut microbiota, which may serve as a focus guide for future ND therapeutic research.

## Methods

This systematic review was reported according to the Preferred Reporting Items for Systematic Reviews and Meta-Analyses (PRISMA) guidelines.

### Literature search

A literature search was conducted to identify all existing articles, up to August 2021, that were related to the role of ketogenic diet on the gut microbiota in the context of neurodegeneration. The search terms “Ketogenic diet,” “High fat diet,” “Gut microbiome,” “Gut microbiota,” “Neurodegeneration” and other synonym terms to the previous terms were used in three electronic databases; PubMed, Ovid MEDLINE, and SCOPUS. A title, abstract and keyword search was performed using the search terms. The Boolean operator “AND” was used to link the terms together on all databases. Articles were first screened through their titles and abstracts before proceeding with full text screening of relevant articles.

### Literature selection

The Preferred Reporting Items for Systematic Reviews and Meta-Analysis (PRISMA) guideline was employed during the literature selection (Moher et al., 2015). The following inclusion criteria were used during the selection process; (1) peer-reviewed original research articles investigating the interplay between ketogenic diet, gut microbiome/microbiota and neurodegeneration and (2) English articles with full text available. The exclusion criteria were; (1) non-original research articles labeled as editorials, symposiums, conference papers, commentaries, book chapters, case reports, systematic reviews, and reviews, (2) duplicated articles, (3) articles that were not in the English language, and (4) articles that did not investigate ketogenic diet effects on gut microbiota in the context of neurodegeneration.

### Quality appraisal

Different tools were employed to evaluate the quality of the selected relevant articles. The Quality Assessment Tool for Quantitative Studies by the Effective Public Health Practice Project (EPHPP) (Project, 1998) was used to assess the clinical studies. The Systematic Review Center for Laboratory Animal Experimentation Risk of Bias (SYRCLE RoB tool) was used to assess the quality of the preclinical animal studies. The literature search, selection and quality analysis of the selected articles were performed by two independent researchers.

## Results

**Figure 2:**
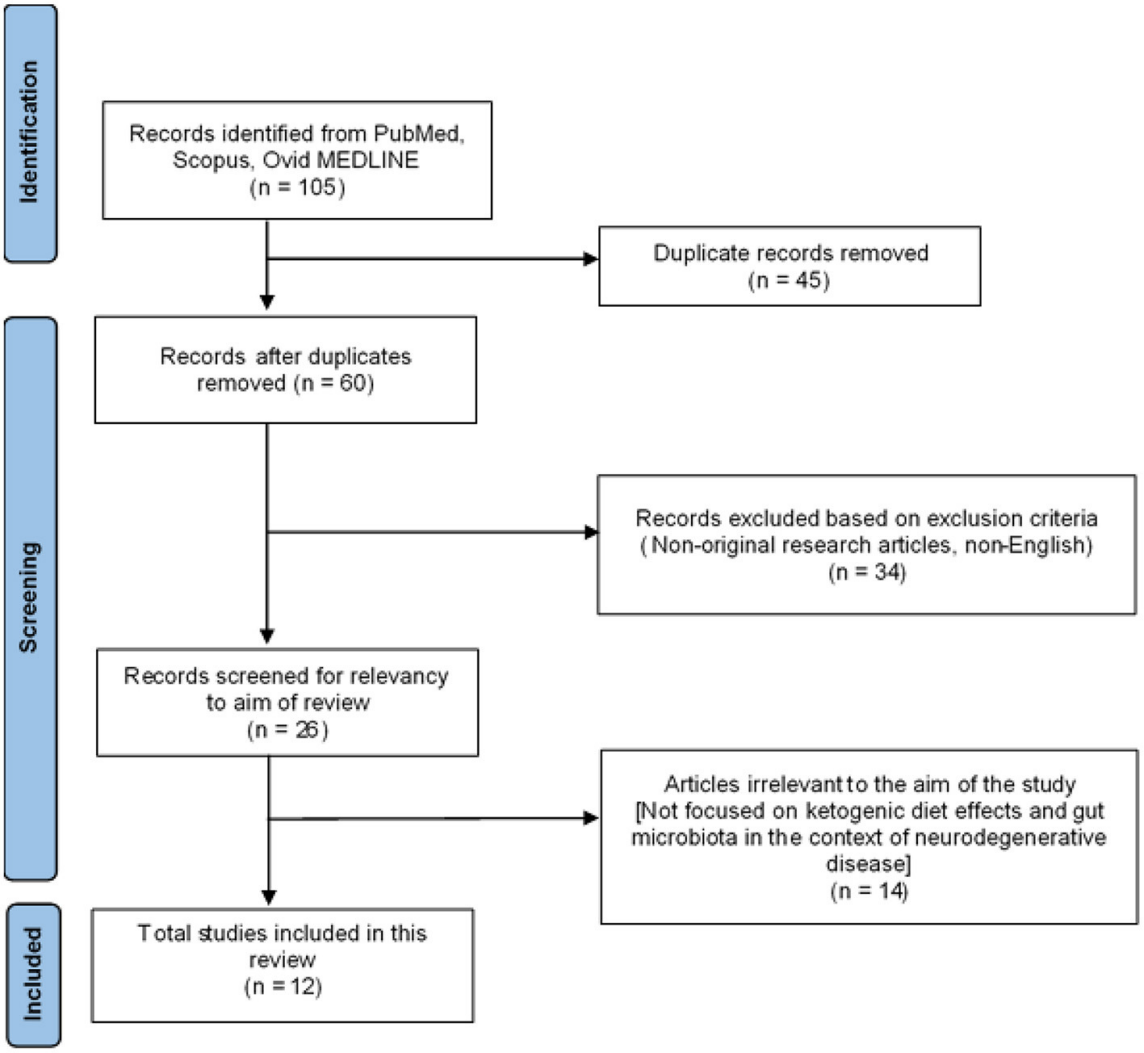
PRISMA flowchart diagram.

The initial literature search retrieved a total of 105 articles collectively from the three databases: 29 from PubMed, 20 from Ovid MEDLINE and 56 from SCOPUS. Based on the inclusion and exclusion criteria, 45 duplicated articles were removed. The remaining 60 articles were screened in accordance with the PRISMA guidelines (Figure 2). A total of 34 articles were then excluded as they were not original research articles, not available in full text or not in the English language. The remaining 26 original research articles were screened for relevancy to the aim of this systematic review; the effects of ketogenic diet on gut microbiota in the context of neurodegeneration/neurodegenerative disease. This led to the exclusion of 14 studies, which were found irrelevant to the aim of this review; not involving the interplay between ketogenic diet, neurodegeneration, and gut microbiota. Thus, 12 studies were finally included in this systematic review for critical appraisal (Figure 2). These studies were further categorized into different neurodegenerative diseases and further subdivided into clinical (three articles) and preclinical animal studies (nine articles) within each category. Table 1 summarized the significant findings from these articles. Most of the preclinical studies adopted a ketogenic/high-fat diet in which 60% of calories originate from fat, 20% from protein and carbohydrate each.

**Table 1:** Summary of study characteristics and significant findings of ketogenic diet in association with gut microbiota and neurodegenerative disease.

**ND**	**Type of study**	**Sample type, Age and Gender/Strain**	**Ketogenic diet (composition, duration)**	**Significant finding: Gut microbiota and ND profile**		**References**
				**Baseline in disease condition**	**After keto**	
**Mild Cognitive Impairment (MCI)**	Clinical (RCT)	11 MCI, 6 CN (cognitively normal) 64.6 ± 6.4 years old 12 females, 5 males	**Modified Mediterranean style ketogenic diet (MMKD)** ° <10% carbohydrate ° 60–65% fat ° 30–35% protein 6 weeks	• No significant difference between baseline alpha and beta diversity • Lower: Genus *Meyerozyma* correlated positively with AB40 and total tau • Higher: Genera *Botrytis, Kazachstania, Phaeoacremonium, Cladosporium, Aspergillus*	• (Insignificant) Increase in alpha diversity. • Decrease: Genera *Botrytis, Hannaella* • Genera *Aspergillus* and *Cladosporium* formed a negative correlation with p-tau • Distinct co-occurrence between fungal and bacterial groups	Nagpal et al. (2020)
**Mild Cognitive Impairment (MCI**)	Clinical (RCT)	11 MCI, 6 CN (cognitively normal) 64.6 ± 6.4 years old 12 females, 5 males	**Modified Mediterranean style ketogenic diet (MMKD)** ° <10% carbohydrate ° 60–65% fat ° 30–35% protein 6 weeks	• No significant difference between baseline alpha and beta diversity • Higher: a) Phyla Proteobacteria*, Firmicutes, Tenericutes. - Firmicutes has a positive correlation with tau-p181 - Proteobacteria has a positive correlation with Aß42/Aß40 ratio b) Families Enterobacteriaceae* Mogibacteriaceae* - *Enterobacteriaceae* has a positive correlation with tau-p181 and tau-p181/Aß42 ratio,	• Decrease a) Family Bifidobacteriacea* b) Genus *Lachnobacterium* • Increase: a) Phyla Tenericutes, Verrucmicrobia - Tenericutes has a negative correlation with Aß42 b) Genera *Akkermansia, Slackia*	Nagpal et al. (2019)
				- *Mogibacteriacea* has a positive correlation with Aß42/Aß40 ratio c) Genera *Coprococcus, Phascolarctobacterium* • Lower: Phyla Bacteriodetes, Verrucomicrobia		
**Multiple Sclerosis (MS)**	Clinical (RCT)	24 MS, 14 controls (unknown age, and gender distribution)	**Ketogenic diet (KD)** ° <50 g carbohydrate °>160 g fat ° <100 g protein 6 months	• Lower: - Diversity* of all substantial group by 36% - Concentration* of all substantial group by 24% • (Slightly) Higher: - Cor653, Cvir1414, Ehal, Ecyl387, Lab158, Rfla729	• Increase: Essential and individual substantial grou • Decrease: Individual pioneer group and *Akkermansia*	Swidsinski et al. (2017)
**Alzheimer's Disease (AD)**	Preclinical (Controlled Trial)	50 male rats (40 AD, 10 Non-AD) unknown age Sprague-Dawley strain	**Ketogenic diet, AD-KD** ° 0.1 En% carbohydrate ° 81 En% fat ° 19 En% **Intermittent fasting, AD-IMF** ° 0.1 En% carbohydrate ° 81 En% fat ° 19 En% protein 8 weeks	**Control diet, AD-CON** • Higher Order Clostridales • Increase B-amyloid deposition • Increase in mRNA expression of TNF-a, IL-1b within hippocampus • Decrease in Akt phosphorylation • Decrease in FOXO-1 phosphorylation	**AD-KD** • Significant decrease in number of bacterial species. • Decrease Shannon Index compared to AD-CON • Increase: Phylum Proteobacteria (Enterobacteriales) • Increase in B-amyloid deposition and tau phosphorylation • Decrease in Akt phosphorylation **AD-IMF** • Decrease: *Clostridales*, • Increase: *Lactobacillales*.	(Park et al., 2020)
					• Increase in Akt and FOXO1 phosphorylation • Decrease in B-amyloid deposition, and tau phosphorylation	
**Alzheimer's Disease (AD)**	Preclinical trial	28 juvenile Iberian pigs 13 Days old	**Ketogenic diet regimen** ° 10 g fructose ° 20.6 g fat ° 314.8 kcal of ME **High Fat High Fructose (HFF) diet regimen** °+6.2*10^4^ cfu/mL probiotics 10 weeks	Not Applicable	• Increase in genera *Bacteroides, Bilophila, Synergistes, Kopriimonas, Bulleidia*, and *Olsonella* which positively correlates with astrocyte reactivity marker GFA • Genus *Sharpeo* was also found to be elevated, coinciding with a negative correlation to marker of number of mature neurons NeuN alongside genus *Olsonella*.	Zeltser et al. (2020)
**Alzheimer's Disease (AD)**	Preclinical trial	52 male mice (40 AD, 12 Non-AD) 3 month old APP/PS1 double-transgenic mice and Parasite-free wild-type mice	**Ketogenic diet regim** ° 60% Fat ° 20% Carbohydrate ° 20%Protein 6 months	Not Applicable	Decrease in genera *Akkermansia* abundance in the APP/PS1 transgenic mice with age.	Ou et al. (2020)
**Alzheimer's Disease (AD)**	Preclinical trial	28 male mice (12 AD, 16 Non-AD) 3 months old 5xFAD heterozygous mice	**Ketogenic diet regi** ° 60% Fat ° 20% Carbohydrate ° 20%Protein 4.5 months	Not applicable	Increase in genera *Firmicutes* and a decrease of *Bacteroidetes* and *Actinobacteria* were noted across both WT and 5xFAD groups after prescription of HFD	Reilly et al. (2020)
**Neurodegeneration (Neurological Diseases)**	Preclinical trial	40 male mice 8 weeks old C57BL/6 mice	**Ketogenic diet regim** ° 60% Fat ° 20% Carbohydrate ° 20%Protein 10 weeks	Not Applicable	Increase in *Bilophila sp*. and a decrease in genera *Akkermansia* was observed	Bruce-Keller et al. (2015)
**Neurodegeneration (Neurovascular Diseases)**	Preclinical trial	18–20 healthy male mice 12–14 weeks of age C57BL/6 mice	**Ketogenic diet regimen** ° 3.2% carbohydrates ° 75.1% fat ° 8.6% protein 16 weeks	Not Applicable	• Decrease in species diversity among KD mice • Decrease in genera Desulfovibrio*, Turicibacter*, Clostrodium*, Dorea* • Decrease in mTOR expression. • Increase in *Akkermansia Muciniphila, Lactobacillus* • Increase in cerebral blood perfusion to VMH region, eNOS levels, P-glycoprotein expression and activity levels	Ma et al. (2018)
**Non-Alcoholic Steatohepatitis (NASH)**	Preclinical trial	40 male rats 8 weeks old Sprague-Dawley rats	**Ketogenic diet regim** ° 39 g Fat ° 19 g Carbohydrate ° 2 g Cholesterol ° 27 g Protein 14 weeks	Not Applicable	• Decrease in proportion of *Firmicutes*, and an increased proportion of *Bacteroidetes* and *Proteobacteria* were observe • Decrease in family *Ruminococcaceae, Lactobacillaceae*, genus *Lactobacillus, Clostridium cluster IV, Bifidobacterium* • Increase in family *Peptostreptococcaceae, Clostridiaceae, Enterobacteriaceae, Desulfovibrionaceae, Bacteroidaceae*, genus *Bacteroides, Blautia, Rombustia, Bilophila, Escheria-Shigella* were identified	Higarza et al. (2019)
**Amyotrophic lateral sclerosis (ALS)**	Preclinical trial	24 Female rats (14 SOD1 and 10WT) 10 weeks old B6.Cg-Tg(SOD1*G93A)1Gur/J mice	**Ketogenic diet regim** ° 60% Fat ° 20% Carbohydrate ° 20%Protein 80 days	Not Applicable	• Decrease in family *Rikenellaceae, Akkermansiaceae, Gastranaerophilales* and *gamma-proteobacteria(with etomoxir)* were identifie • Increase in family *Lachnospiraceae* and *Odoribacter*	Trabjerg et al. (2021)

MCI, Mild cognitive impairment; CN, Cognitively normal; MMKD, Modified Mediterranean-style Ketogenic Diet; MS, Multiple Sclerosis; KD, Ketogenic Diet; AD, Alzheimer's Disease; IMF, Intermittent fasting; CON, Control; eNOS, endothelial Nitric oxide synthase; NCD, Normal chow diet; HFF, High-fat high-fructose diet; HFD, High-fat diet; AKKsub, Akkermansia muciniphila subtype; C57BL/6, C57 black 6 mice; APP/PS1, APP and PS1 double transgenic mice; 5xFAD, Transgenic mice with 5 familial AD mutations.

## Discussion

This systematic review highlighted that neurodegenerative diseases, such as MCI, AD, and ALS have an altered gut microbiota (gut dysbiosis), which predominantly consisted of increases in harmful phyla such as Firmicutes and Proteobacteria, as well as decreases in beneficial phyla like Verrucomicrobia (Akkermansia). This review also elucidated the contrasting effects of ketogenic diet on the ND gut dysbiosis, which were dependent on the duration of the diet, the type of ND and possibly the influence of environmental factors.

### Alteration in baseline gut microbiota in the disease model

There were no significant differences seen between the baseline alpha and beta diversity of the gut microbiota among the MCI participants compared to cognitively normal participants (Nagpal et al., 2019, 2020). On the other hand, MS participants depicted an overall significant decline in bacterial diversity by 36% and a bacterial mean total concentration by 24% compared to the healthy subject (Swidsinski et al., 2017). In addition, the data collected by Swidsinski et al. connotated that patients with multiple sclerosis have an underlying colonic dysbiosis due to the diminished biodiversity and concentration of the essential bacterial groups, such as Faecalibacterirum prausnitzii (Fprau) (Swidsinski et al., 2017). Fprau has been categorized as an anti-inflammatory microbe due to its capacity to generate butyrate (Bhargava and Mowry, 2014; Cantarel et al., 2015), which may play a central role in sustaining balance and maintaining the optimal ratio of regulatory T cells (T-reg) and T helper 17 cells (Th17) (Bhargava and Mowry, 2014; Cantarel et al., 2015). Hence, disruption to this balance may, in turn, induce inflammation (Bhargava and Mowry, 2014; Cantarel et al., 2015), thereby possibly leading to aggravation of the neurodegeneration condition.

Taxonomical evaluation of the gut microbiota illuminated a predominance of phyla Proteobacteria and Firmicutes amongst patients with underlying ND. There was a significant elevation in the phyla Proteobacteria in fecal samples of subjects with MCI in which the bacteria positively correlated with an AD-biomarker, Aß42/Aß40 ratio (Nagpal et al., 2019). Furthermore, family *Enterobacteriaceae* (phylum Proteobacteria) was also significantly elevated among MCI subjects and correlated positively with AD-biomarker tau-p181 and tau p-181/ Aß42 ratio (Nagpal et al., 2019). In Alzheimer's disease, high abundance of *Enterobacteriaceae* was also associated with worsening of disease progression and decline in cognitive functioning. Notably, patients that harbored a high proportion of Proteobacteria had significantly low Mini Mental State Examination (MMSE) and Montreal Cognitive Assessment (MoCA) (Liu et al., 2019).

Similarly, phyla Firmicutes was also elevated amongst those with MCI at baseline and positively correlated with AD-biomarker tau p-181 (Nagpal et al., 2019). Patients with MS were found to have an elevated abundance trend of rRNA bacteria Cvir1414 (*Clostridium viridae* group), Ehal (*Eubacterium hallii*), Ecyl387 (*Eubacterium cylindroides*), Lab158 (*Lactobacillus, Enterococcus sp*.), and Rfla729 (*Ruminococcus albus*) upon colonic microbiota analysis *via* fluorescence in-site hybridization method (FISH), whereby all these bacterial groups belong to the phylum Firmicutes, (Swidsinski et al., 2017) but these elevation was statistically insignificant. In accordance with this, the Sprague Dawley rats also demonstrated a relatively higher abundance of order Clostridales which belongs to the phylum Firmicutes in the AD group (Park et al., 2020).

### Influence of ketogenic diet on microbiota

Two studies depicted that a ketogenic diet had resulted in a decrease in bacterial species diversity. For example, the rats within the AD-ketogenic diet group depicted a significant decline in bacterial species and a decrease in the Shannon Index (diversity) (Park et al., 2020). The finding by Park et al. aligns with an earlier study by Ma et al. in which introduction of a ketogenic diet regime to healthy mice resulted in decreased species diversity (Ma et al., 2018). A similar pattern of enrichment of gut bacterial α-diversity was observed in mice treated with etomoxir, as well as in mice with genetically modified lower CPT1 activity known as SOD1^Cpt1a/Cpt1a^. However, when these mice were assigned a 60% high fat diet, they showcased a drop in the α-diversity. Hence, it is believed that the downregulation of CPT1 in ND models by ketogenic diet may decrease the gut microbiota diversity, which may attenuate disease progression. Furthermore, a loss of α-diversity was also seen after the commencement of high-fat diet (Bruce-Keller et al., 2015; Higarza et al., 2019), where a diminished proportion of Firmicutes, and an augmented proportion of Bacteroidetes and Proteobacteria was observed in the high-fat diet group (Higarza et al., 2019). Additionally, a decrease in family Ruminococcaceae, Lactobacillaceae, genus *Lactobacillus, Clostridium* cluster IV, *Bifidobacterium*, and an increase in family Peptostreptococcaceae, Clostridiaceae, Enterobacteriaceae, Desulfovibrionaceae, Bacteroidaceae, genus *Bacteroides, Blautia, Rombustia, Bilophila, Escheria-Shigella* were all identified in the high-fat diet group.

Similarly, another study showed an increase in phylum Bacteroidetes, genera *Bacteroides, Dorea, Roseburia, Bilophila* and a fall in size of phyla Actinobacteria, Cyanobacteria, Tenericutes, genera *Bifidobacterium, Odoribacter, Dehalobacterium, Turibacter*, after high-fat diet (Bruce-Keller et al., 2015; Wu et al., 2020). However, no specific pattern of changes in gut microbiota corresponding to the duration of dietary interventions were reported in these studies. In addition, differential abundance testing revealed a significant drop in harmful bacterial genera *Rikenellaceae, Akkermansiaceae, Gastranaerophilales*, and ⋎*-proteobacteria* in the group treated with etomoxir. Remarkably, class ⋎*-proteobacteria* and family Rikenellaceae have been widely held as pro-inflammatory bacteria, with the latter being linked to disruption of intestinal epithelial integrity and exacerbation of colonic inflammation *via* toll-like receptor (TLR)4 signaling pathway (Liu et al., 2019).

There is a pronounced increase in beneficial bacterial genera *Lachnospiraceae* and *Odoribacter* in the SOD1^Cpt1a/Cpt1a^ mice group, as compared to their respective control counterparts. *Odoribacter*, has been known to generate lower levels of the pro-inflammatory cytokine, tumor necrosis factor-alpha (TNF-α) (Trabjerg et al., 2021). This increase in beneficial bacterial composition was also seen with the genus *Akkermansia*, where after ketogenic diet intervention, the genus *Akkermansia* was elevated in MCI subjects (Nagpal et al., 2019) and even in healthy mice (Ma et al., 2018). It was also shown that in a high-fat diet animal model, the cognitive decay was halted, and spatial memory was improved by increasing the kynurenic acid production with the help of *Akkermansia* (Wu et al., 2020). *Akkermansia* was also reported to relieve systemic inflammation by decreasing the concentration of pro-inflammatory markers such as IL-10 and increasing anti-inflammatory markers (Wu et al., 2020). Furthermore, *Akkenmansia* was also depicted to restore gastrointestinal ecosystem by increasing the concentration of genus *Bifidobacterium*, neuroprotective bacteria, and decreasing the neurotoxic genera *Bilophila* and *Bacteroidetes* (Wu et al., 2020). In addition, *Akkermansia* may also produce beneficial SCFA, predominantly acetate and propionate, which was shown to be vital for gut and neuronal communication and homeostasis (Wu et al., 2020). Taken together, this evidence suggest that ketogenic diet not only reduced harmful bacterial composition, but also promoted the flourishment of beneficial bacterial species such as *Akkermansia*. In addition, harmful bacteria such as genus *Lachnobacterium* (of phylum Firmicutes) was found to be downregulated in patients with MCI after ketogenic diet intervention (Nagpal et al., 2019). Similarly, prominent down-regulation of pro-inflammatory bacteria genera Desulfovibrio and Turicibacter were also observed in mice on ketogenic diet (Ma et al., 2018). In fact, the concentration of Desulfovibrio was undetectable, whereas Turicibacter was two times lower in concentration compared to the control mice. Likewise, positive gut microbiota modulation was seen with ketogenic diet intervention, whereby the bacterial composition in patients with MS, was restored and almost coincided with the healthy control subjects after the diet (Swidsinski et al., 2017).

### Influence of ketogenic diet on clinical impairment

Mice that were on the ketogenic diet demonstrated enhanced abundance of the beneficial bacteria genus *Lactobacilli* by 3.2 times (Ma et al., 2018). The increase in *Lactobacilli* and the decline in *Desulfovibrio* by ketogenic diet provided a shift within the internal gut environment to an anti-inflammatory state (Solas et al., 2017; Leigh and Morris, 2020). In support, the administration of probiotics containing *Lactobacillus* demonstrated restoration of cognitive impairment, whereas feeding of chow containing *Desulfovibrio* was shown to perpetuate the cognitive decline (Leigh and Morris, 2020), thus suggesting the positive impact of ketogenic diet on these two bacterial genera and possibly on neurodegeneration as well. The data collected by Swidsinski et al. connotated that patients with MS have an underlying colonic dysbiosis due to the diminished biodiversity and concentration of the essential bacterial groups, such as Faecalibacterirum prausnitzii (Fprau) (Swidsinski et al., 2017). Fprau has been categorized as an anti-inflammatory microbe due to its capacity to generate butyrate (Bhargava and Mowry, 2014; Cantarel et al., 2015) which may play a central role in sustaining balance and maintaining the optimal ratio of regulatory T cells (T-reg) and T helper 17 cells (Th17). Hence, disruption to this balance may, in turn, induce inflammation, thereby possibly leading to aggravation of the neurodegeneration condition (Bhargava and Mowry, 2014; Cantarel et al., 2015).

### Influence of ketogenic diet on others

Ketogenic diet has the ability to diminish the oxidative stress in neurodegenerative diseases, particularly MS, by increasing the levels of glutathione and catathione, which are enzymes that may exert antioxidant properties and counteract the increasing levels of reactive oxygen and nitrogen species, respectively (Tobore, 2021). Mice on the ketogenic diet intervention have also demonstrated enhanced global cerebral perfusion, whereby regional blood flow to the ventromedial hypothalamus (VMH) was significantly increased by 11.82% (Ma et al., 2018). In addition, Aβ clearance was also augmented in the mice under ketogenic diet (Ma et al., 2018). From a molecular perspective, the ketogenic diet reduced the production of mTOR protein by dampening its gene expression by 29.9%. Inhibition of mTOR protein expression may aid in delaying cognitive deterioration, as hyperactivation of this protein has been associated with memory impairment (Jahrling and Laberge, 2015). Besides that, endothelial nitric oxide synthase (eNOS) levels were also found to be significantly boosted by 111.5% increase in the ketogenic diet mice (Ma et al., 2018). eNOS has been seen as a potent vasodilator, and hence increased circulating levels of eNOS by ketogenic diet may promote cerebral perfusion especially to the hypothalamus (Liao et al., 2021). A trial comprising of eNOS deficient mice models depicted pathological signs suggestive of the neurodegenerative process such as loss of white matter mass leading to cortical atrophy, along with decreased concentration of oligodendrocytes, resulting in lack of myelin sheaths (Liao et al., 2021). Hence, this elucidated the vital function of eNOS in maintaining a healthy CNS environment. In addition, the expression of P-glycoprotein, an essential protein that may aid Aβ clearance from the brain, was also significantly elevated by 50% in ketogenic diet mice and its activity was promoted by 185.38% (Ma et al., 2018). The study by Park et al. further elucidated that integration of ketogenic diet as an intermittent diet schedule have proven to be more favorable, where a decline in Firmicutes and an augmentation in Akt phosphorylation were observed (Park et al., 2020). Upstream signaling of the Akt molecule causes the activation of a cascade of molecular processes, which ultimately may lead to dampening of the AD pathogenesis, such as decreased Aβ deposition and formation of neurofibrillary tangles (Long et al., 2021).

### Negative aspect of ketogenic diet

Some studies have suggested that ketogenic diet exerts a negative effect on the gut microbiota. The conflicting results indicate that ketogenic diet as a therapy for gut dysbiosis in neurodegeneration may warrant further investigations, particularly in a clinical setting, where influences of environmental factors and inter-species interactions in the intestinal flora may govern the therapeutic effects of ketogenic diet. A surge in abundance of family Streptococcaceae and Desulfovibrionaceae, which have been known for their pro-inflammatory and endotoxin-producing properties, as well as a decrease in the family Lachnospiraceae have been reported after a high-fat diet regime suggesting a inflammatory mediating effect of high-fat diet (Li et al., 2021; Trabjerg et al., 2021). Additionally, signs of gut dysbiosis have been observed in the AD rat group after ketogenic intervention, where there was an increase in the order Enterobacteriales (phylum Proteobacteria) (Park et al., 2020). Furthermore, the ketogenic dietary pattern also resulted in a further increase in *Enterobacteriaceae* from baseline among MCI subjects (Nagpal et al., 2019). Likewise, diminutions of the family *Bifidobacteriacea* (of phylum Actinobacteria) upon ketogenic diet in MCI patients also depicted an unfavorable outcome of this diet pattern in the neurodegeneration context (Nagpal et al., 2019). In addition, within the MS population, a decline in levels of *Akkermansia* was observed after six months of ketogenic intervention (Swidsinski et al., 2017), unlike the positive increase seen in MCI subjects (Nagpal et al., 2019).

Similarly, in an ALS transgenic animal model receiving high fat diet, worsening of neurological score, earlier onset of tremor, weaker grip strength, reduced overall activity level, and lesser right turns in Y-maze test were reported further exemplifying the diet's possible deleterious consequences in ALS (Trabjerg et al., 2021). Correspondingly, from the pathological aspect, a heightened state of neuroinflammation was also shown by the rise in astrocyte reactivity marker, microglial marker (*Cx3cr1* and *Tmem119*), TLR 2&4, and the decrease in ZO-1 and claudin 5, which are indicative of compromised neurovascular integrity, in subjects receiving the high fat diet (Bruce-Keller et al., 2015; Reilly et al., 2020; Zeltser et al., 2020). High fat diet was also found to render intestinal tight junction more permeable (lower jejunum and colonic occluding, increased DAO), thereby inducing a state of systemic inflammation, exemplified by increased colonic NOS, phosphorylation of p65 subunit of NFKD, plasma endotoxin and the pro-inflammatory IL-1β (Bruce-Keller et al., 2015; Trabjerg et al., 2021). In fact, Wu et al. also showed that a high-fat diet promoted memory decay and depression in mice, suggesting that a high-fat diet may indeed be more harmful than beneficial (Wu et al., 2020). Besides that, the contrasting behavioral and pathological effects seen between ketogenic diet intervention and high fat diet intervention may be related to the state of carbohydrate deprivation, which is higher in ketogenic diet than high fat diet. Thus, based on the evidence accumulated in this systematic review, ketogenic diet may be more beneficial toward neurodegenerative diseases than high fat diet, especially when integrated as an intermittent diet schedule, but the duration of the diet should be monitored closely. There is a substantial amount of evidence that proposes the positive molecular modulation of ketogenic diet in a healthy environment, but whether these positive modulations may persist in a neurodegeneration model may warrant further investigations.

## Conclusion

Neurodegenerative disorders may lead the development of gut dysbiosis. This systematic review has highlighted the effects of ketogenic diet in modulating the interaction between the gut microbiota and the pathological pathway in neurodegenerative diseases. Ketogenic diet has shown to restore/promote beneficial microbes such as *Akkermansia* and confer neuroprotection by enhancing perfusion to the brain parenchyma, increasing amyloid-beta clearance and decreasing pro-inflammatory cytokines. Furthermore, cognitive functioning was also shown to be regulated by ketogenic diet, with improvements in memory, learning and spatial visualization. However, the conflicting/contrasting results demonstrated by ketogenic diet, such as a decline in gut species richness, diminution in beneficial microbes and decline cognition unless delivered in an intermittent fasting pattern, may warrant further investigations in neurodegenerative disease models prior to recommendation of a ketogenic diet therapeutic regime in ND patients.

## Data availability statement

The original contributions presented in the study are included in the article/supplementary material, further inquiries can be directed to the corresponding author.

## Author contributions

TR, AA, and MS have conceptualized, designed the study, and revised manuscript critically. SK and EC performed the literature search and drafted the manuscript. TR, SK, and EC collected and assembled the data. SK, EC, TR, AA, and MS analyzed the articles. All authors read and approved the submitted manuscript version.

## Conflict of interest

The authors declare that the research was conducted in the absence of any commercial or financial relationships that could be construed as a potential conflict of interest.

## Publisher's note

All claims expressed in this article are solely those of the authors and do not necessarily represent those of their affiliated organizations, or those of the publisher, the editors and the reviewers. Any product that may be evaluated in this article, or claim that may be made by its manufacturer, is not guaranteed or endorsed by the publisher.
